# Research on the Earth Pressure and Internal Force of a High-Fill Open-Cut Tunnel Using a Bilayer Lining Design: A Field Test Using an FBG Automatic Data Acquisition System

**DOI:** 10.3390/s19071487

**Published:** 2019-03-27

**Authors:** Tianyuan Xu, Mingnian Wang, Li Yu, Cheng Lv, Yucang Dong, Yuan Tian

**Affiliations:** Key Laboratory of Transportation Tunnel Engineering, Ministry of Education, Southwest Jiaotong University, Chengdu 610031, Sichuan, China; 19910622@163.com (M.W.); lvchengaust@163.com (C.L.); yucang_dong@my.swjtu.edu.cn (Y.D.); ytian_ty@163.com (Y.T.)

**Keywords:** high-fill open-cut tunnel, bilayer lining, FBG sensors, automatic monitoring system, earth pressure of open-cut tunnel

## Abstract

When there are railway tunnels on both sides of a valley, a bridge is usually built to let trains pass. However, if the valley is very close to an urban area, building an open-cut tunnel at the portal and then backfilling it to create available land resources for the city and to prevent excavation slag from polluting the environment would be a wise choice. This has led to the emergence of a new type of structure, namely, the high-fill open-cut tunnel. In this paper, by performing an automatic long-term field test on the first high-fill open-cut tunnel using a bilayer design in China, the variations of earth pressure and structural internal force during the backfilling process were obtained, and different tunnel foundation types were studied. The results showed that the earth pressure significantly exceeded the soil column weight, with a maximum earth pressure coefficient between 1.341 and 2.278. During the backfilling process, the earth pressure coefficient increased at first and then decreased slowly to a relatively stable value, and a stiffer foundation would make the structure bear higher earth pressure (1.69 times the normal one observed during monitoring). The change of internal force had two stages during backfilling: before the backfill soil reached the arch crown, the internal force of the lining changed slowly and then grew linearly as the backfill process continued. Moreover, the axial force ratio of the inner and outer linings was close to their thickness proportion, and the interaction mode between the two layers was very similar to the composite beam.

## 1. Introduction

**Figure 1:**
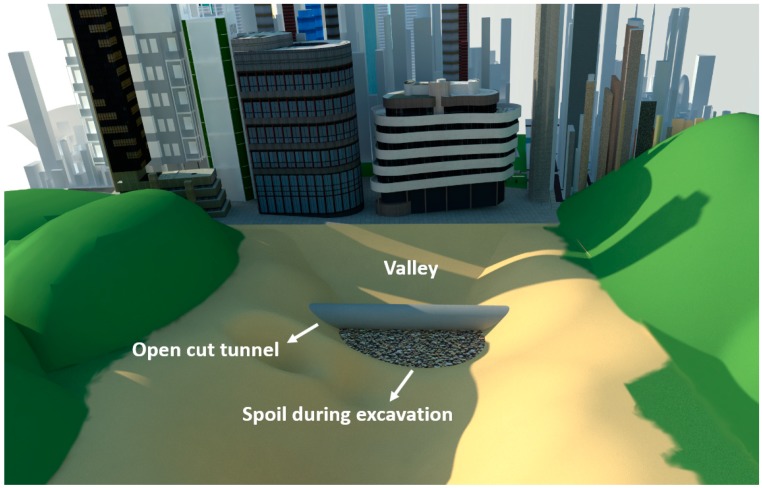
Open-cut tunnel built in a valley close to a city.

In China, the high-speed railway network is rapidly extending into the mountainous area of the southwest. Due to the geological conditions of the southwest region, there has been a substantial increase in the number of tunnels needed, some of which are near cities. There is a special situation for some of these cities: sometimes, there is a valley between two tunnels that is very close to urban areas. In a normal situation, a bridge must be built above the valley to let trains pass. However, if an open-cut tunnel were built at the portal and then backfilled with the engineering spoils from the tunnel excavation, available land resources can be created for cities, which would prevent waste from polluting the environment and lead to the emergence of high-fill open-cut tunnels, as shown in Figure 1. Compared with the traditional open-cut tunnels, a high-fill open-cut tunnel has the following two characteristics:

(1) The height of backfill layer can reach up to 30–40 m, which is almost five times larger than a common one;
(2) In order to bear such high earth pressure, the thickness of the tunnel lining can reach up to 2 m or more, which is 4–5 times thicker than those with shallow overburden. Details of existing high-fill open-cut tunnels in China are shown in Table 1.

These two characteristics present two research difficulties:

(1) A large amount of hydration heat can be generated after construction if a monolithic design is adopted for these superthick concrete linings, causing a significant temperature difference between the internal and external surfaces of the lining, which would result in structural shrinkage cracks and safety issues. Therefore, a more reasonable structure design must be adopted.

In order for the lining structure to meet the bearing capacity requirement for such a high backfill layer and to minimize the shrinkage cracks at the same time, a bilayer design is used for high-fill open-cut tunnels which divides the overthick lining into two parts—outer and inner linings. Two layers are concreted separately at 14-day intervals, resulting in a significant decrease of hydration heat generated by each layer of lining. However, the bilayer lining design initially appeared in shield tunnels, which consist of segmental and secondary linings, and has never been used in open-cut tunnels.

Yang et al. [1] carried out a numerical study on the performance of segmental and secondary linings as well as the stress transmission between them. They reported that linings with rebar had a combined bearing capacity, while linings with membranes had separate bearing capacities. Vogel et al. [2] examined double-shell linings in respect to direct shear stress capacity. Their results revealed that a spray-applied waterproofing membrane was able to transfer stresses between both concrete linings but could not transfer any shear stress. Su and Bloodworth [3] presented laboratory tests on beam samples cut from bilayer linings. They compared the influence of different membrane thicknesses and substrate roughness and provided parameters based on test results for further research and design. Their subsequent research [4] developed a composite mechanical behavior quantification method. Chuan et al. [5] presented several load tests on bilayer shield tunnel models, the results of which revealed that the secondary lining bore most of the bending moment. Their following study focused on a calculation model for bilayer linings [6].

**Figure 2:**
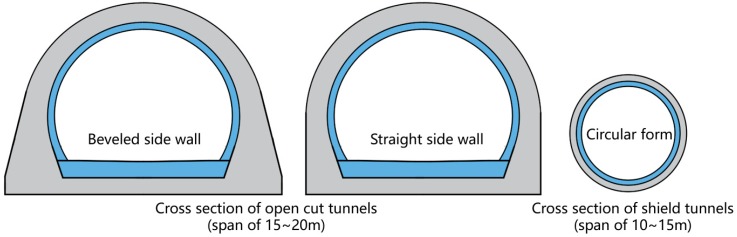
Comparison of size and shape of open-cut and shield tunnels using the bilayer design.

The abovementioned studies provide a better understanding of the mechanical characteristics of the bilayer lining tunnel design, as well as the calculation model and load shearing ratio needed to design the structure. However, the construction sequence of open-cut tunnels is completely the opposite of that of shield tunnels, in which the lining is built at last. Moreover, the size and shape of open-cut tunnels are also quite different from shield tunnels (circular design), as shown in Figure 2.

(2) Under such a high backfill layer, it still remains unclear whether the earth pressure on the tunnel crown equals the overburden pressure. Moreover, a deep foundation has been adopted for high-fill structures in some situations, which can also affect the earth pressure of the structure. 

A high-fill situation usually occurs in culverts and pipelines in mountainous areas and has never been adopted in railway open-cut tunnels. Qiang et al. [7] analyzed several calculation methods of earth pressure on a culvert crown. The results showed that under a high embankment fill condition, the calculated results by the “neutral point” method were closer to the values measured in situ. Li et al. [8] carried out a field monitoring and numerical simulation to investigate the state and distribution of stress on the exterior surface of slab-culverts under high embankments. The results showed that the earth pressure on culverts was larger than the self-weight of filling, and the pressure distribution was uneven. Chen et al. [9] presented a new formula to calculate the vertical earth pressure on a culvert, the calculation of which results were compared with field tests. Zhang et al. [10] carried out a centrifugal experiment on the earth pressure distribution of a culvert top. The results showed that a box culvert with a pile foundation bore higher earth pressure than a normal one, and they suggested that soil could be backfilled first on both sides of the culvert before it was constructed. Some researchers developed a load calculation method for deeply buried culverts by summarizing the displacement distribution characteristics on the culvert crown [11,12]. Additionally, controlling displacement was considered to reduce the vertical pressure on culverts. Moreover, backfill materials and some engineering measures also affect the earth pressure of high-fill structures. Meguid [13] presented an experimental investigation to measure the earth pressure distribution on a rigid pipe backfilled with tire-derived aggregate. The average measured earth pressure above the crown of the pipe was found to be as low as 30% of the overburden pressure. An optimum soft zone geometry for imperfect trench installation was proposed to maximize the reduction of the earth pressure on buried corrugated steel pipes [14], and the maximum wall stress was reduced by 69%.

According to previous research, the earth pressure of an open-cut structure is closely related to the side slope angle and foundation type. As a more rigid foundation usually results in a higher earth pressure, it is suggested to avoid using a deep foundation in the design of culverts. However, some culverts still use a strengthened foundation to achieve better performance in particular situations [15]. For railway open-cut tunnels, the track surface subsidence must be strictly controlled to meet the operation requirements of high-speed trains, making it necessary to use deep foundations for railway open-cut tunnels in poor geological conditions. In conclusion, a high-fill open-cut tunnel using a bilayer lining design is very different from the current culvert or shield tunnel in terms of construction method, shape, size, and foundation type, and its mechanical characteristics are not clear yet. Besides, current research rarely contains long-term field measurement data, especially measurements conducted when the structure is built on different types of foundations.

In geotechnical and structural domains, fiber Bragg grating (FBG) sensors are applied to measure structural strain [16,17,18,19], seepage pressure [20,21], temperature [22], and vibration [23]. However, the monitoring work for tunnels does not continue after the lining has stabilized. Therefore, vibrating wire sensors are more widely used to reduce unnecessary costs, and these sensors typically require manual data acquisition. However, for dynamic or long-term data acquisition from tunnels, such as the real-time safety monitoring of tunnels in special geological conditions and measuring the vibrations generated by train travel [24,25], it is more sensible to use FBG sensors, as they have strong anti-interference and long-term stability advantages. In our research, FBG pressure cells and strain gauges were adopted to acquire the soil pressure and structural internal force for further analysis.

Moreover, for an open-cut tunnel, once the backfilling process begins, personnel have to enter the tunnel from the undercut portal, which sometimes is more than 10 km from the test section. Therefore, an automatic data acquisition system was adopted to provide more timely and accurate data. These test results can be a useful complement to both high-fill open-cut and bilayer structures to help provide a deeper understanding of them.

## 2. Field Test Procedure

### 2.1. Tunnel Description

The field test was carried out at the first high-fill open-cut tunnel in Fengdu, China, where the bilayer lining design was firstly adopted, with a 0.5-m-thick C35 concrete inner lining covered by a 1.4–2.8-m-thick C35 concrete outer lining. Further, a waterproof layer consisting of nonwovens and a polyethylene sheet was applied between the inner and outer linings as well as on the surface of the outer lining. The outer lining was constructed first. When the outer lining was stabilized, the inner lining was constructed, and finally, the backfilling process was carried out.

**Figure 3:**
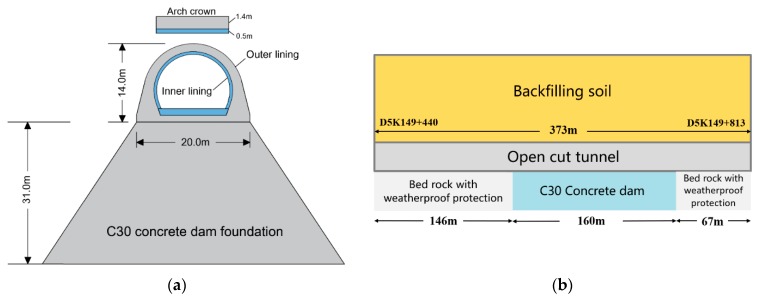
Design details of the tunnel. (**a**) Cross section of the tunnel and the concrete dam foundation; (**b**) Profile view of the whole project.

**Figure 4:**
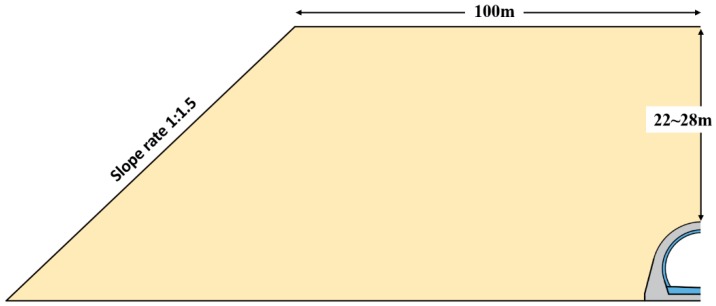
Backfilling range (half side).

**Figure 5:**
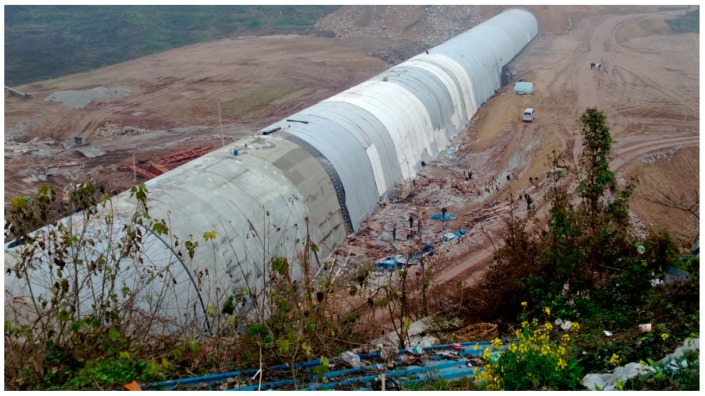
Open-cut tunnel in the backfill process.

This open-cut tunnel is 373-m long with a backfill height up to 22–28 m (from tunnel arch crown to the backfill surface) and a 100-m horizontal backfill range on both sides of the tunnel axis. Both sides of the tunnel were filled symmetrically with 0.5-m-thick earth in each cycle, which took 6–7 days, and the whole backfill process lasted about 9 months. Also, a C30 concrete dam was adopted as the foundation for some part of the open-cut tunnel to control the subsidence of the tunnel bottom. The remaining parts of the open-cut tunnel were set on bedrock with weatherproof protection. More design details are shown in Figure 3, Figure 4 and Figure 5.

### 2.2. Automatic Data Acquisition System

The application of intelligent test systems and methods in tunnels has been a growing trend in recent years [26,27,28,29]. In our field test, an automatic data acquisition system was developed and adopted, using FBG sensors to measure the structural internal force and soil pressure. The system was developed by the research team in collaboration with Sensorlead Technology Co., Ltd, Shanghai, China. It can capture the wavelength data of the sensors at 100 Hz and allows users to input different formulas to convert wavelength data into stress, pressure, displacement, etc. Moreover, it has a remote operating subsystem to allow users to view the data anytime by personal computers or even smartphones.

**Figure 6:**
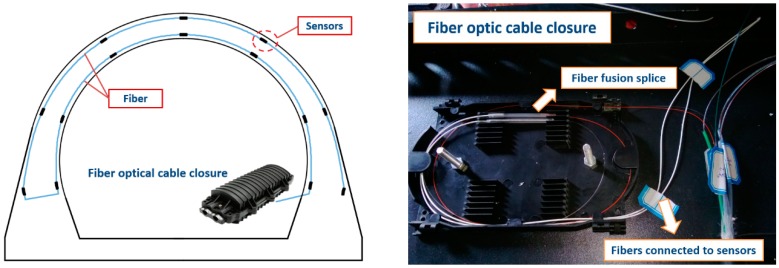
Fiber optic cable closure.

Step 1: Adjacent sensors were connected by optical fibers at both ends and were finally connected to a fiber optic cable closure, as shown in Figure 6.

Step 2: All the closures were connected in a series and were finally connected to one main fiber cable.

**Figure 7:**
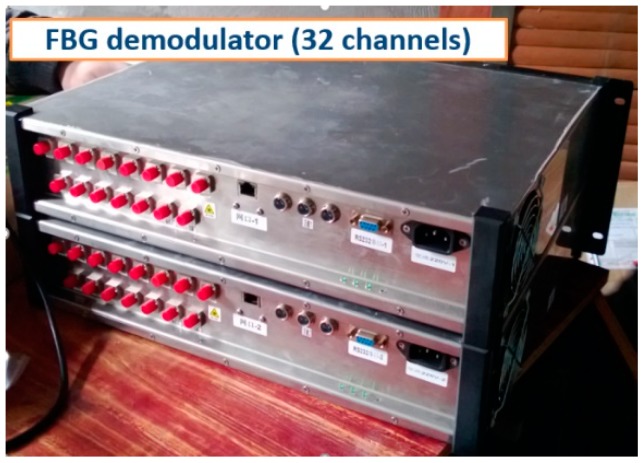
FBG demodulators.

**Figure 8:**
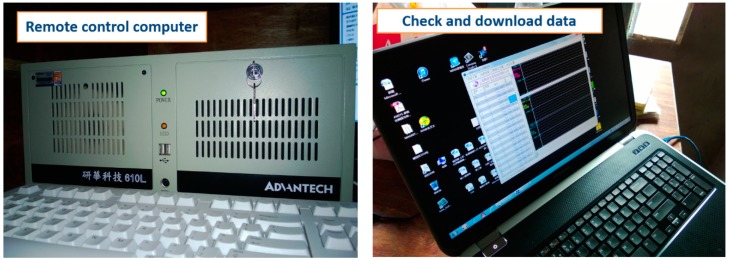
Remote-control computer.

**Figure 9:**
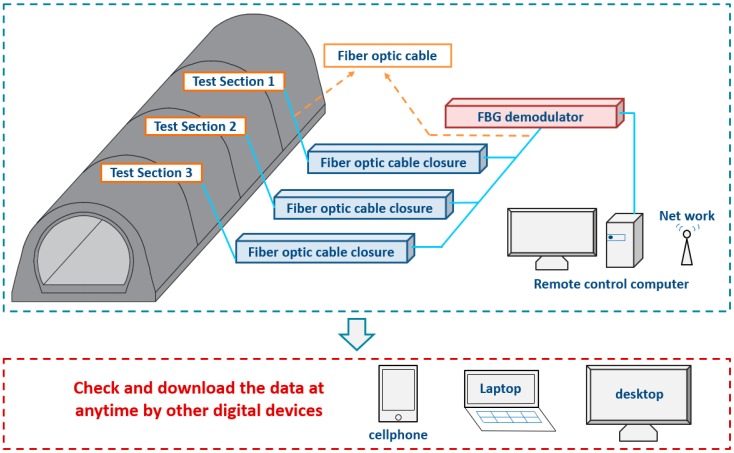
Automatic data collecting and delivering system.

Step 3: The main fiber cable was connected to FBG demodulators (Figure 7), which transmitted the data to a remote-control computer, as shown in Figure 8 and Figure 9.

Figure 9 shows the flow chart of the whole data acquisition system. In order to reduce the test error caused by construction disturbance, the data acquired at 4:00 a.m. was used for subsequent analysis.

### 2.3. Sensor Layout and Basic Structure

Two sections were chosen to test the earth pressure and structural internal force, using bedrock and the concrete dam as their foundation, respectively, as shown in Table 2.

**Figure 10:**
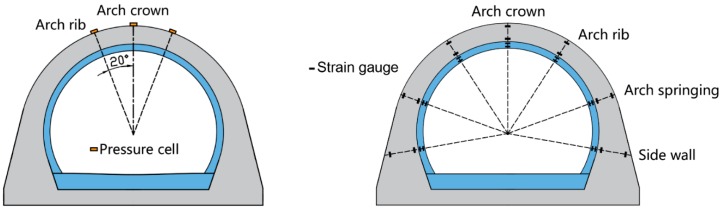
The layout of pressure cells and strain gauges.

**Figure 11:**
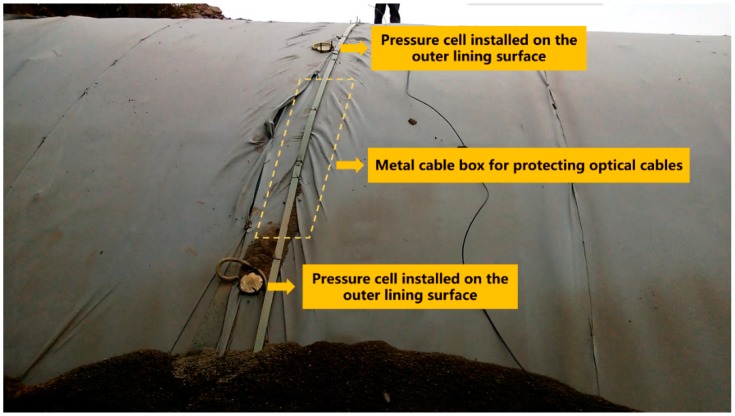
Pressure cells on the outer lining surface with metal cable boxes to protect the optical cables.

In order to obtain the variation law of earth pressure and structural internal force during the backfilling process, earth pressure cells were installed at the arch crown and arch rib on the outer lining surface, and strain gauges were installed in the inner and outer linings, respectively, as shown in Figure 10. For those pressure cells installed on the outer lining surface, metal cable boxes were used to protect the fiber cables from being damaged by the falling backfill soil, as shown in Figure 11.

**Figure 12:**
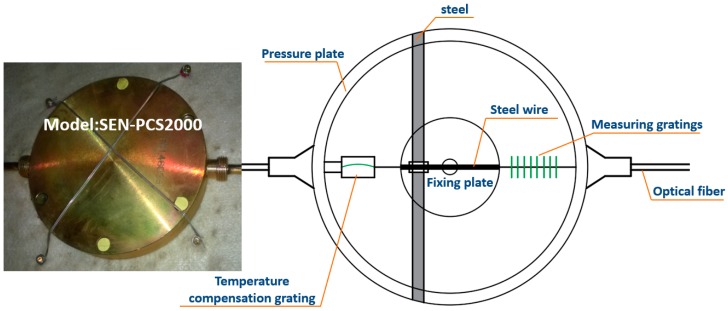
Model and basic structure of pressure cells.

**Figure 13:**
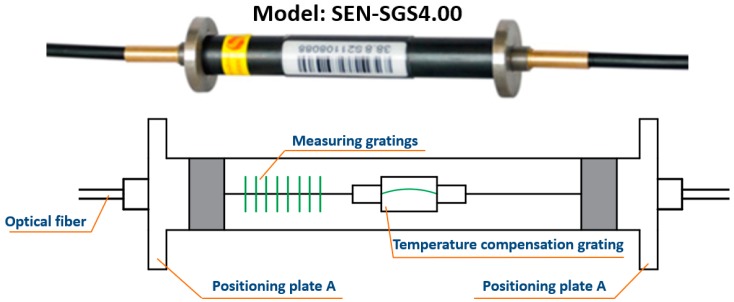
Model and basic structure of gauges.

The gauges and cells adopted in this research were made by Sensorlead Technology Co., Ltd. Shanghai, China. Their model and basic structure are shown in Figure 12 and Figure 13. Changes in stress and pressure can cause changes in the wavelength of the sensor, which could be measured by the gratings. Further, real-time ambient temperature could be read by the temperature compensation gratings in the sensors, correcting errors due to seasonal temperature differences and providing more accurate data.

## 3. Numerical Model

**Figure 14:**
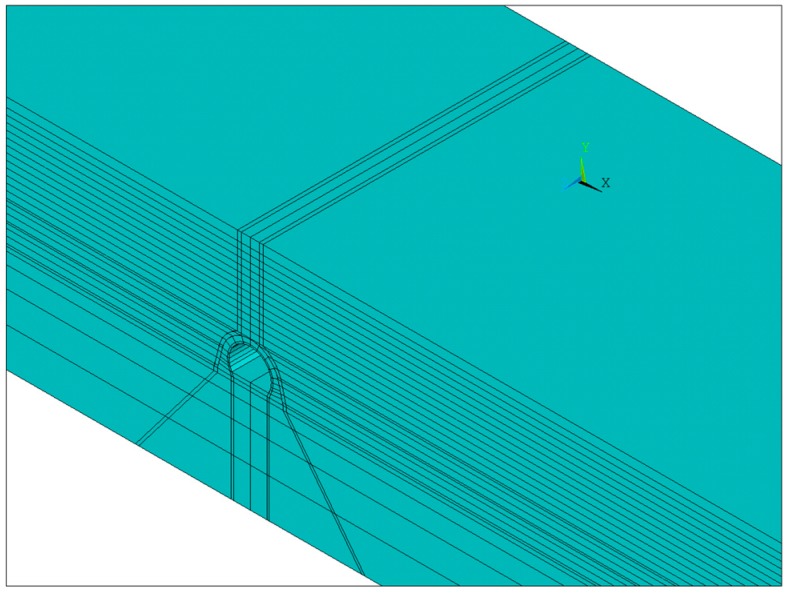
Three-dimensional finite element model for a numerical simulation from ANSYS 12.0 software.

**Figure 15:**
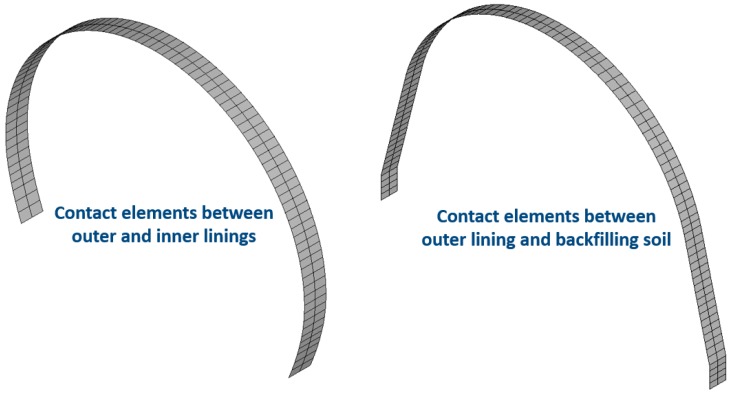
Contact elements between different structures.

For further study and design improvement of a high-fill open-cut tunnel using bilayer linings, a numerical model was built in this study, as shown in Figure 14. The elastic model was adopted for the lining and concrete foundation of the open-cut tunnel, and the Drucker–Prager elastic–plastic model was used for the bedrock and backfill soil. The contact elements were added between the open-cut tunnel and the backfill to simulate the mutual squeezing and slip between them (Figure 15). According to the site situation, a waterproof layer consisting of nonwovens and a polyethylene sheet was set between the outer lining and backfill soil as well as between the outer and inner linings. The friction coefficient between the nonwovens and the polyethylene sheet was 0.23. Moreover, the backfilling process of the open-cut tunnel was simulated by the method of layered activation, that is, finite element units for every single backfilling layer (0.5 m) were activated sequentially. The calculation parameters of the model are shown in Table 3.

**Figure 16:**
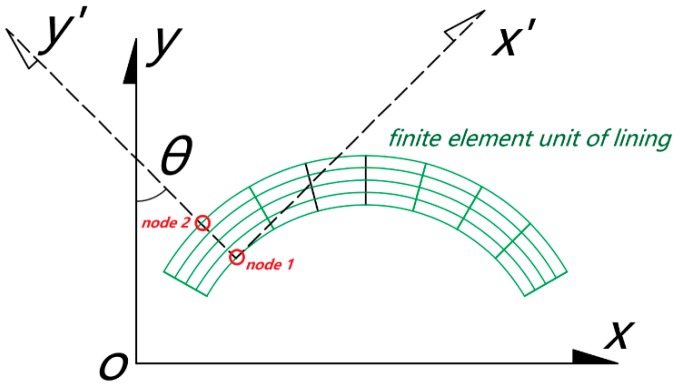
Coordinate transformation for node stress of lining unit.

In the 3-D numerical model, the node stress in the original Cartesian coordinate system was transformed into the tangential stress of the lining by the coordinate transformation of elastic mechanics. As a result, the bending moment and axial force of the lining were obtained and used for further analysis. The transformation method is described below (Figure 16).

Taking nodes 1 and 2 on the outer and inner sides of a section, and assuming that the angle between the line of the two nodes above and the vertical plane is *θ*, the tangential stress of each node on the lining section can be calculated from Equation (1) [30]:

(1) $$\sigma_{n}=\sigma_{x}\cos^{2}\theta +\sigma_{y}\sin^{2}\theta +\tau_{xy}\sin2\theta$$

where $\sigma_{x}$, $\sigma_{y}$, and $\tau_{xy}$ are node stress components in the original coordinate system, respectively, and θ is the angle between the outer normal of the section and the *y* axis.

Therefore, the tangential stress of nodes 1 and 2 was obtained, and then the axial force *N* and bending moment *M* of the lining section could be deduced according to Equations (2) and (3) [30]:

(2) $$M=bh^{2}\frac{\sigma_{1}-\sigma_{2}}{12}$$

(3) $$N=bh^{}\frac{\sigma_{1}+\sigma_{2}}{2}$$

## 4. Earth Pressure Test Results and Discussion

### 4.1. Earth Pressure Test Results

**Figure 17:**
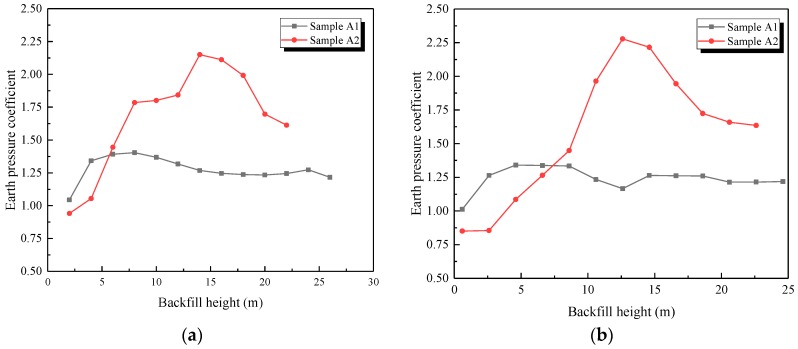
Variation of earth pressure coefficient during the backfilling process. (**a**) Arch crown; (**b**) Arch rib.

The in situ earth pressure was obtained in the backfilling process of the open-cut tunnel. The measured results are represented by the earth pressure coefficient (*c*), which was defined as earth pressure divided by bulk density, as described in Equation (4). The average earth pressure of the left and right arch rib acted to reduce the testing error. The testing results are shown in Figure 17:

(4) $$c=\frac{p}{\rho gh}$$

where *p* is the earth pressure, *ρ* is the soil density, and *h* is the backfilling height.

**Figure 18:**
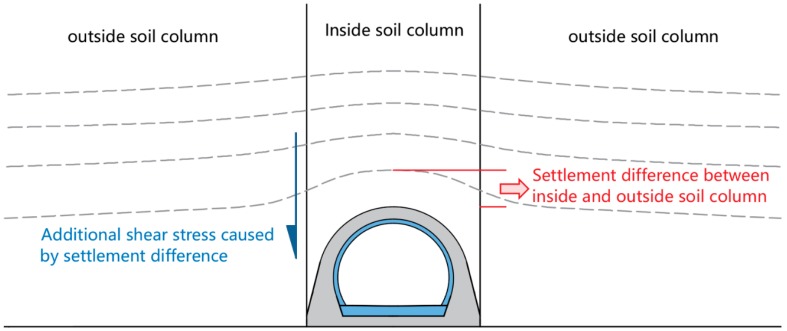
Additional shear stress caused by the settlement difference between inside and outside soil columns.

As shown in Figure 17:

(1) The earth pressure coefficient firstly increased and then decreased slowly with the increasing backfilling height. The earth pressure coefficient of sample A1 reached its peak value when the backfilling height was around 8 m and then decreased slowly until stabilizing. However, the maximum coefficient of sample A2 appeared at the stage when the backfilling height was about 14 m and then rapidly decreased until stabilizing. The different change trends of the earth pressure coefficient between samples A1 and A2 means that the interaction process in the soil was gradual during the backfilling phase. 

(2) At same backfilling height, the earth pressure coefficient at the arch crown and arch rib of sample A2 was obviously higher than that of sample A1, respectively, as listed in Table 4. The earth pressure coefficient at the arch crown of sample A2 was 1.53 times that of sample A1, and sample A2 was 1.69 times that of sample A1 at the arch rib. The root cause of this phenomenon was the additional shear stress difference between samples A1 and A2. The additional shear stress was caused by the settlement difference between the inside and outside soil columns, as shown in Figure 18. The inside soil column consisted of lining and the above soil. Its stiffness was much higher than that of the outside backfilled soil, which led to the settlement difference between the outside and inside soil columns. So, the outside soil column pulled the inside soil column down, resulting in additional shear stress, as shown in Figure 18. Therefore, compared with the stiffness of sample A1, that of sample A2 was much higher, resulting in greater differential settlement and, finally, a significant increase in earth pressure.

In summary, at the same backfilled height, the increasing stiffness and thickness of the base caused there to be higher earth pressure acting on the open-cut tunnel, which increased the bearing capacity requirement for the lining. Nevertheless, rail surface settlement must be strictly controlled to satisfy the operation requirements of railway trains. Thus, a deep foundation is always applied in open-cut railway tunnels which cross through weak and soft ground. So, to avoid excessive loading of the lining, load shedding measures can be used which have already been adopted in high-fill culverts and pipes [31]. For instance, the load shedding layer was installed above the structure to decrease the settlement difference between the inside and outside soil columns, which reduced the loading of the open-cut structure [32].

### 4.2. The Comparative Analysis of In Situ Testing and Numerical Simulation 

**Figure 19:**
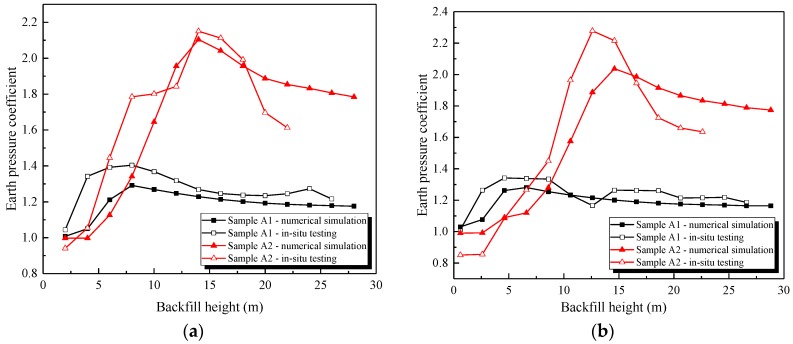
Earth pressure coefficient (comparison of numerical simulations and in situ testing). (**a**) Arch crown; (**b**) Arch rib.

In order to validate the abovementioned conclusion, a comparative analysis of the in situ test and numerical simulation is shown in Figure 19.

As shown in Figure 19, the earth pressure coefficient change trend of the numerical simulation was similar to that of the in situ test, which firstly increased and then decreased until stabilizing. Moreover, the vertical earth pressure coefficient of sample A2 was obviously higher than that of sample A1, as listed in Table 5. The earth pressure coefficient at the arch crown of sample A2 was 1.63 times that of sample A1, and sample A2 was 1.59 times that of sample A1 at the arch rib.

The in situ testing results were in good agreement with the numerical simulation results, and the maximum error was 21.7% at the beginning phase of the backfilling process. The reason for this could be that the backfill had not been completely compacted and stabilized during the actual construction.

In summary, both the in situ testing and the numerical simulation demonstrated that the total loading that acted on the high-fill open-cut tunnel consisted of two parts: the additional loading and the soil column loading. Moreover, the value of additional loading was closely related to the stiffness of the base. In order to more accurately determine the earth pressure of a high-fill open-cut tunnel, the lining and foundation below must be considered as a whole structure. The results also verified the accuracy of the numerical model, which can be used to analyze the settlement difference between the inside and outside soil columns, as shown in Section 4.3.

### 4.3. The Settlement Difference Analysis between the Inside and Outside Soil Columns

**Figure 20:**
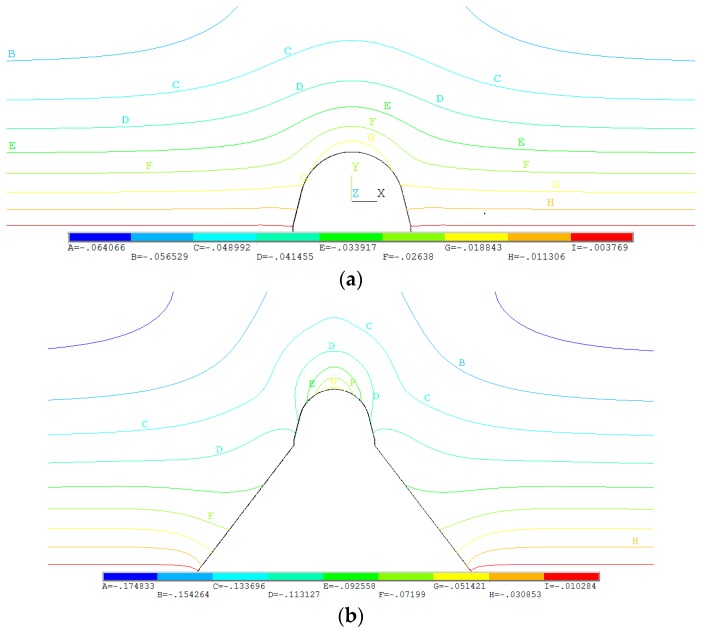
Settlement difference between the inside and outside soil columns. (**a**) Settlement difference for sample A1; (**b**) Settlement difference for sample A2.

The key factor for the additional loading was the settlement difference between the inside and outside soil columns. However, the change trend or results of this difference could not be obtained exactly through in situ measurement, making it difficult to explain the gradual decreasing phenomenon of the earth pressure coefficient. Therefore, the relationship between earth pressure and settlement difference was analyzed via numerical simulation. The settlement difference between the inside and outside soil columns of two base types is illustrated in Figure 20.

**Figure 21:**
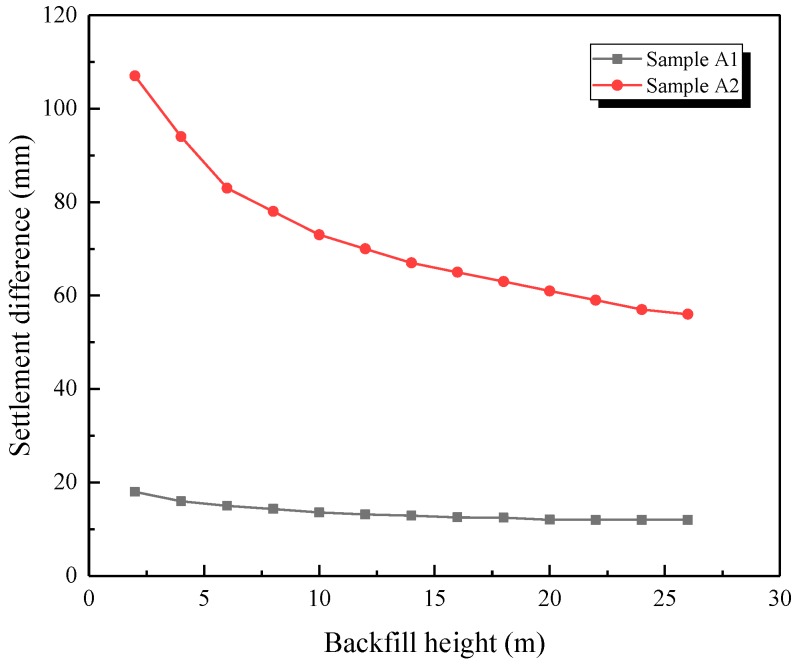
Settlement difference variation law as the backfill height increased.

As presented in Figure 20, the settlement difference of sample A2 was obviously higher than that of sample A1. The settlement difference results from the arch crown in the 2–28-m range are shown in Figure 21, where the settlement difference on the horizontal plane near the arch crown for sample A1 was 18 mm, while that of sample A2 was 107 mm, about six times that of sample A1, which means the stiffness of the base had a significant influence on the settlement difference. Moreover, the settlement difference gradually decreased to a stable value with the increasing backfill height, which clearly explains the change trend of the earth pressure coefficient.

## 5. Internal Force Test Results and Discussion

**Figure 22:**
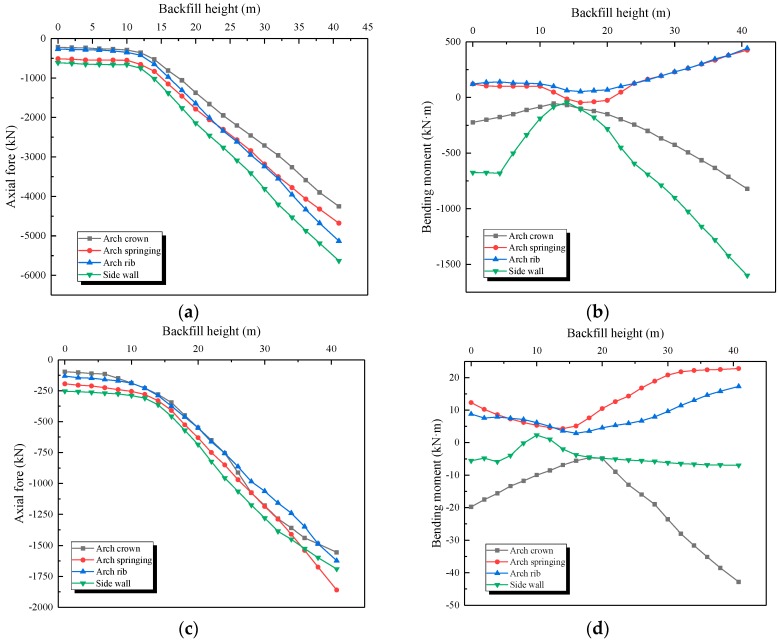
Variation of internal force during the backfilling process for sample A1. (**a**) Axial force of outer lining (sample A1); (**b**) Bending moment of outer lining (sample A1); (**c**) Axial force of inner lining (sample A1); (**d**) Bending moment of inner lining (sample A1).

**Figure 23:**
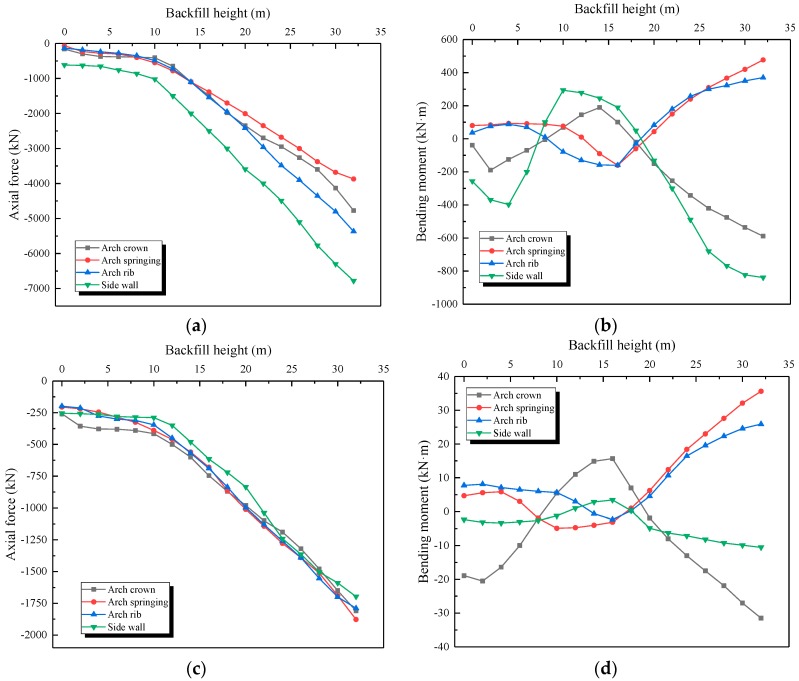
Variation of internal force during the backfilling process for sample A2. (**a**) Axial force of outer lining (sample A2); (**b**) Bending moment of outer lining (sample A2); (**c**) Axial force of inner lining (sample A2); (**d**) Bending moment of inner lining (sample A2).

The structural strain data were firstly transformed into tangential stress by multiplying the elastic modulus of the material. The test data of the measuring points in the symmetrical position were averaged to reduce the error. The dynamic change of axial force and bending moment of the bilayer lining during the backfilling process is shown in Figure 22 and Figure 23.

The change trend of axial force and bending moment can be represented as two stages:

Stage 1: Before the backfilling soil reached the arch crown, the structure only bore lateral pressure, resulting in a very small increase of the axial force and bending moment.

Stage 2: When the backfilling soil exceeded the arch crown, the lining bore both lateral and vertical pressure. The axial force showed linear growth, while the bending moment linearly decreased to a negative value. Moreover, the internal force change trend of samples A1 and A2 during the backfilling process was quite similar.

However, the absolute value of the internal force of the tunnel on the concrete dam foundation was much higher than the one without the concrete dam. The axial force of sample A1 was 1.2–1.6 times that of the other, and the bending moment was 1.6–2.4 times that of the other, as seen in Table 6. This further proves that an open-cut tunnel using a concrete dam foundation will bear greater earth pressure at the same backfilling height, and the internal force of the structure will also increase significantly.

In addition, the axial force of the outer lining was about 2.6–3.2 times that of the inner lining, which was very close to their thickness ratio (3:1), as shown in Table 6. This was because the waterproof layer transition between the two linings reduced the friction between the outer and inner linings. The transmission of shear force between the outer and inner linings was blocked, while only the axial force could be transmitted. That is, the mechanical behavior of the double lining could be analyzed according to the combined beam model.

## 6. Conclusions

A field test study was carried out at the first high-fill open-cut tunnel using the bilayer design in China. An automatic data acquisition system using FBG sensors was adopted to obtain the earth pressure and internal force of the tunnel, and numerical models were created for further analysis as well. The following major conclusions can be drawn:

(1) The earth pressure coefficient increased first and then decreased during the backfilling process, and the earth pressure value was significantly higher than the soil column weight. This was because the difference in settlement between the inner and outer soil columns could produce shear forces downward, and the settlement value slowly decreased as the backfill height increased, which was proved by the numerical model above.
(2) At the same backfilling height, the open-cut tunnel on the concrete dam foundation bore greater earth pressure than the one on bedrock, and the internal force of the tunnel on the concrete dam also significantly increased compared with the other one. The lining and the foundation below must be considered as a whole structure to more accurately determine the earth pressure.
(3) The change in axial force and bending moment had two stages, and the boundary point was when the soil reached the arch crown. Before that, the axial force and bending moment increased very slowly. When the soil exceeded the arch crown, an obvious linear growth of the absolute value of the internal force was observed.
(4) Because of the low friction coefficient between the inner and outer linings, the transmission of shear force between them was blocked, while the axial force could be transmitted smoothly, indicating that the mechanical behavior of the double lining was quite similar to the combined beam model.

## Figures and Tables

**Table 1:** Details of three existing open-cut tunnels in China.

Project Name	Backfill Height—From Tunnel Crown to Backfill Surface (m)	Lining Thickness (m)
Lanyu open-cut tunnel	40	1.8–2.4
Longdongbao open-cut tunnel	33	2.8–3.0
Fengdu open-cut tunnel	28	1.9–3.3

**Table 2:** The measuring sections.

Type	Backfill Height	Foundation Form
**Sample A1**	28.0 m	Bedrock with weatherproof protection
**Sample A2**	22.0 m	C30 Concrete dam

**Table 3:** The physical and mechanical parameters of the model.

Object	Elastic Modulus	Density	Poisson Ratio	Cohesion	Internal Friction Angle
Backfill	12.6 MPa	2100 kg/m^3^	0.4	0.1 MPa	22°
Bedrock	7.25 GPa	2300 kg/m^3^	0.32	0.2 MPa	31°
Lining	33.5 GPa	2500 kg/m^3^	0.2		
Dam	31.5 GPa	2500 kg/m^3^	0.2		

**Table 4:** The earth pressure coefficients of different samples.

Position	Sample A1	Sample A2	Times (A2/A1)
Arch crown	1.404	2.15	1.53
Arch rib	1.341	2.278	1.69

**Table 5:** The earth pressure coefficients of different samples in numerical simulations.

Position	Sample A1	Sample A2	Times (A2/A1)
Arch crown	1.292	2.104	1.63
Arch rib	1.281	2.037	1.59

**Table 6:** Relationship between the internal force and the tunnel foundation.

Object	Outer/Inner Lining	Foundation Type	Arch Crown	Arch Rib	Arch Springing	Side Wall
Axial force (kN)	Outer lining	Bedrock	−2960.13	−3552.58	−3504.25	−4200.27
		Concrete dam	−4772.29	−5367.43	−5807.45	−6781.54
	Inner lining	Bedrock	−1286	−1158	−1289	−1386
		Concrete dam	−1810	−1789	−1878	−1698
Bending moment (kN·m)	Outer lining	Bedrock	−492.94	262.17	512.23	−1025.67
		Concrete dam	−987.53	547.12	918.45	−1775.46
	Inner lining	Bedrock	−28	11.42	21.8	−6.5
		Concrete dam	−68.52	25.89	35.6	−10.53
